# Mechanically stable fibrin scaffolds promote viability and induce neurite outgrowth in neural aggregates derived from human induced pluripotent stem cells

**DOI:** 10.1038/s41598-017-06570-9

**Published:** 2017-07-24

**Authors:** Meghan Robinson, Sarah Douglas, Stephanie Michelle Willerth

**Affiliations:** 1 Biomedical Engineering Program, University of Victoria, 3800 Finnerty Road, Victoria, BC V8W 2Y2 Canada; 2 Department of Mechanical Engineering, University of Victoria, 3800 Finnerty Road, Victoria, BC V8W 2Y2 Canada; 3 Division of Medical Sciences, University of Victoria, 3800 Finnerty Road, Victoria, BC V8W 2Y2 Canada; 4 International Collaboration on Repair Discoveries, University of British Columbia, 818 West 10th Avenue, Vancouver, BC V5Z 1M9 Canada

## Abstract

Recent work demonstrated that 3D fibrin scaffolds function as an effective substrate for engineering tissues from pluripotent stem cells. However, the rapid degradation rate of fibrin remains a major limitation when differentiating human pluripotent stem cells for tissue engineering applications. The addition of crosslinking agents, such as genipin, during the polymerization process increases scaffold stability while decreasing the degradation rate of fibrin. Genipin crosslinking alters the physical characteristics of the fibrin scaffolds, which influences the behaviour of the differentiating cells seeded inside. It also possesses neuritogenic and neuroprotective properties, making it particularly attractive for engineering neural tissue from pluripotent stem cells. Here we show that genipin enhances neuronal differentiation of neural progenitors derived from human induced pluripotent stem cells (hiPSCs) in 2D culture and genipin concentration influences the morphological and mechanical properties of 3D fibrin scaffolds. These mechanically stable genipin-crosslinked fibrin scaffolds support hiPSC-derived neural aggregates and induce neurite outgrowth while remaining intact for 2 weeks as opposed to 5 days for unmodified fibrin scaffolds.

## Introduction

Human pluripotent stem cells can divide indefinitely and adopt the identity of any cell type in the body, giving them the potential to regenerate specific tissue types^1, 2^. These two properties make them an attractive option for treating degenerative diseases and traumatic injuries that occur in the central nervous system^3^. Pluripotent stem cells detect and respond to chemical and mechanical cues from their micro-environment, which direct them to become specialized for their desired role in the body^4^. In 2007, Takahashi and Yamanaka showed that it was possible to reprogram human fibroblasts into a pluripotent state by upregulating the expression of four transcription factors, producing human induced pluripotent stem cells (hiPSCs)^2^. These hiPSC lines can be produced from any individual and can be used to engineer replacement tissue for diseased or damaged organs^5^.

Several research groups (Sakiyama-Elbert, Tuszynski, Oswald) have demonstrated that delivering neural cells derived from stem cells using 3D fibrin scaffolds increases their survival and differentiation in the injured spinal cord^6–11^. Fibrin, a blood derived protein, plays a critical role in coagulation. During this process, the enzyme thrombin cleaves fibrinogen into fibrin monomers that assemble into a fibrous 3-dimentional network with physical properties resembling soft tissue^12–14^. Mechanical characteristics, such as compressive modulus, fiber diameter, pore size, and degradation rate, can be controlled by varying the concentrations of fibrin, thrombin, or the cross-linking agent Factor XIIIa^12, 15^. Fibrin scaffolds last between one and two weeks for *in vitro* and *in vivo* applications^10, 16, 17^, limiting their utility for applications requiring longer culture times such as mature neuronal differentiation of hiPSCs, which can take months^18^. Our group previously engineered 3D fibrin scaffolds for generating neural tissue, consisting predominately of neurons, from mouse iPSCs^19, 20^. Our recent study indicated that hiPSC-derived neural progenitors degraded 3D fibrin scaffolds more rapidly than the mouse derived cells, indicating this formulation was not ideal for use with hiPSCs^21^.

The longevity of fibrin can be increased by adding soluble protease inhibitors like aprotinin to cell culture media^16, 22, 23^, or by enhancing the number of chemical cross-links using agents such as genipin^24–28^. For this study, we investigated the use of genipin, a plant-derived cross-linking agent, that catalyzes covalent bonds between primary amine groups, stabilizing protein based biomaterials^29^. We selected genipin as it promotes neurite outgrowth and survival of N2A (a rat neuroblastoma cell line), and PC12 cells (a rat pheochromocytoma cell line), along with rescuing hiPSC-derived neurons from oxidative stress, desirable properties for engineering neural tissue^30–32^. Other groups have successfully used genipin to make stable fibrin formulations for treating cartilage repair and regeneration^24, 33^. However, the use of genipin to formulate 3D fibrin scaffolds for use with hiPSC-derived neural cells has not yet been explored. As the use of genipin enhances the stability of fibrin scaffold through cross-linking, it changes the mechanical properties of the resulting scaffolds, influencing the behavior of stem cells seeded inside^34–36^. Thus, it is important to mimic the mechanical properties to the tissue being generated. This work first investigated the effect of genipin on human iPSC derived neural progenitor viability and differentiation in 2D culture. Next, the effect of genipin cross-linking on the mechanical and morphological properties of 3D fibrin scaffolds was examined. Finally, we demonstrated that these 3D fibrin-genipin scaffolds promoted the survival and neurite outgrowth of human iPSC derived neural progenitors while remaining intact over a two-week culture period.

## Results

### Genipin induces neurite extension of hiPSC-derived neural progenitors in 2D culture

**Figure 1:**
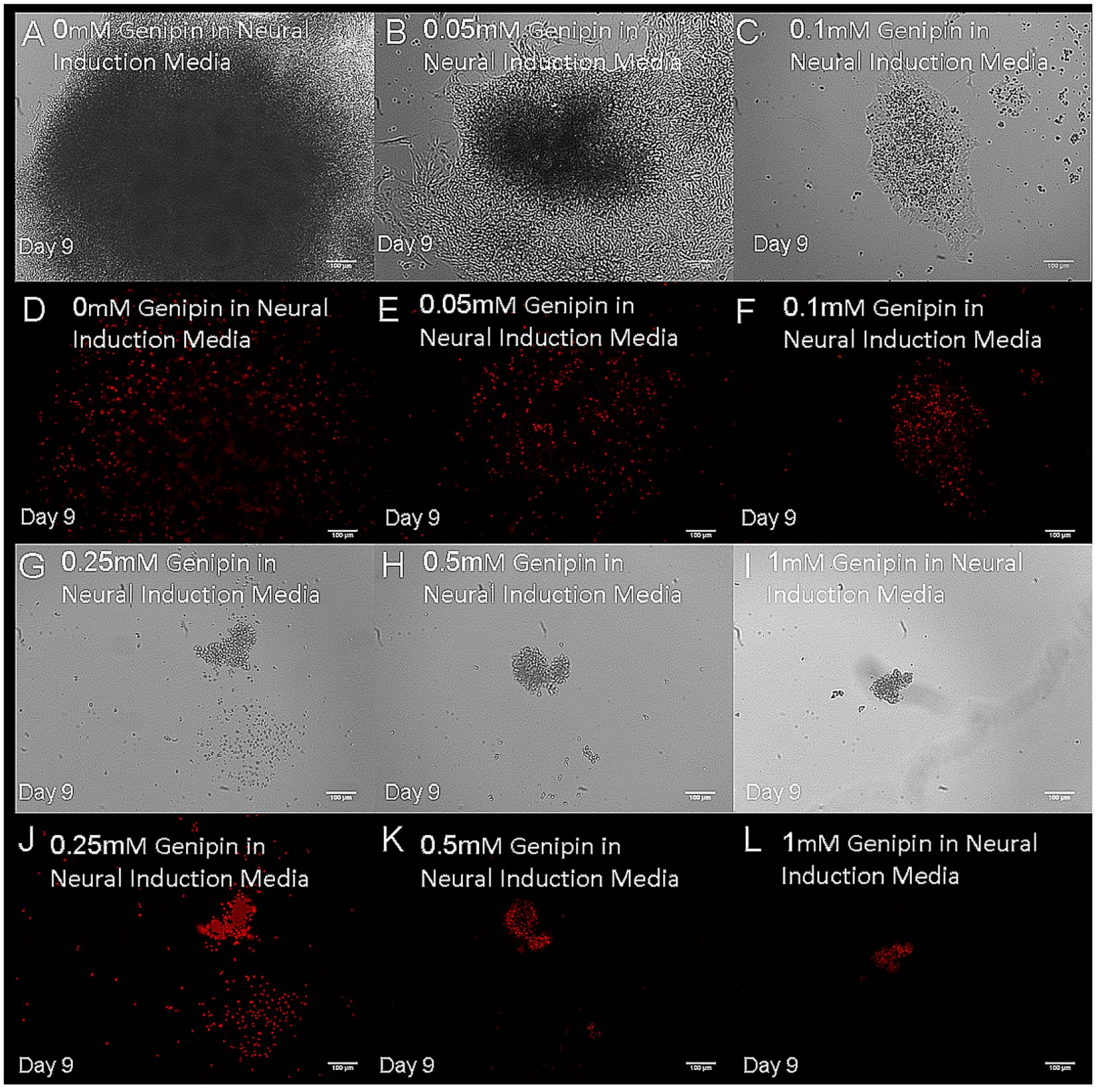
High concentrations of genipin induce death of hiPSC-derived neural progenitors. Images show cell cultures after 9 days of exposure to the following concentrations of genipin: (**A** and **D**) 0 mM, (**B** and **E**) 0.05 mM, (**C** and **F**) 0.1 mM, (**G** and **J**) 0.25 mM, (**H** and **K**) 0.5 mM and (**I** and **L**) 1 mM in phase contrast and fluorescence respectively. Each image represents a single neural aggregate with an initial seeding density of 4000 cells/aggregate (biological n = 3, technical n = 3).

hiPSC-derived neural progenitors seeded on 2D laminin surfaces were treated with a range of genipin concentrations to determine its effect on their behavior. It was previously reported that 0.05 mM genipin induced a neuroprotective and neuritogenic response from Neuro2A cells, and concentrations above 0.05 mM were toxic to these cells^30, 31^. This range of concentrations also corresponds with the amounts of genipin necessary to crosslink fibrin scaffolds. Concentrations above 0.25 mM induced cell death within 24 hours as confirmed by ethidium homodimer staining, a membrane-impermeable fluorescent dye that binds to the DNA of dead cells (Fig. 1). Significantly less cells were observed when higher genipin concentrations were used. The fluorescing dead cells indicate the presence of dead cells, and at high concentrations - no intact neural aggregates were observed.

**Figure 2:**
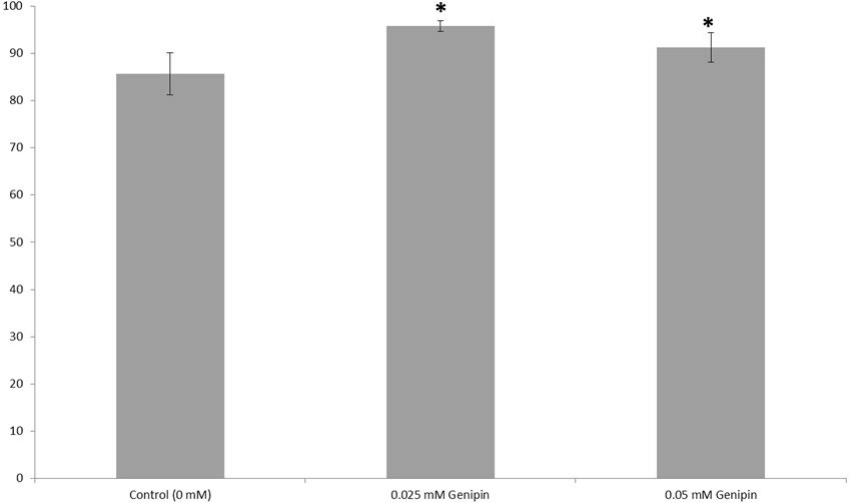
Quantification of viability for hiPSC-derived neural progenitors treated with genipin (biological n = 24, technical n = 5, data reported is mean ± standard error of the mean) *indicates p < 0.5 compared to the control.

The percentage of viable cells in the presence of 0 to 0.05 mM genipin was quantified by flow cytometry with the results being shown in Fig. 2. Cells treated with genipin showed decreased cell death after 8 days compared to control cultures. These results correlate with the observations by Yamazaki *et al*. who found that genipin acts to suppress cytotoxicity in Neuro2A cells by preventing activation of endoplasmic reticulum (ER) stress. It is also hypothesized to induce expression of the genes and proteins that protect against ER stress^31, 33^.

**Figure 3:**
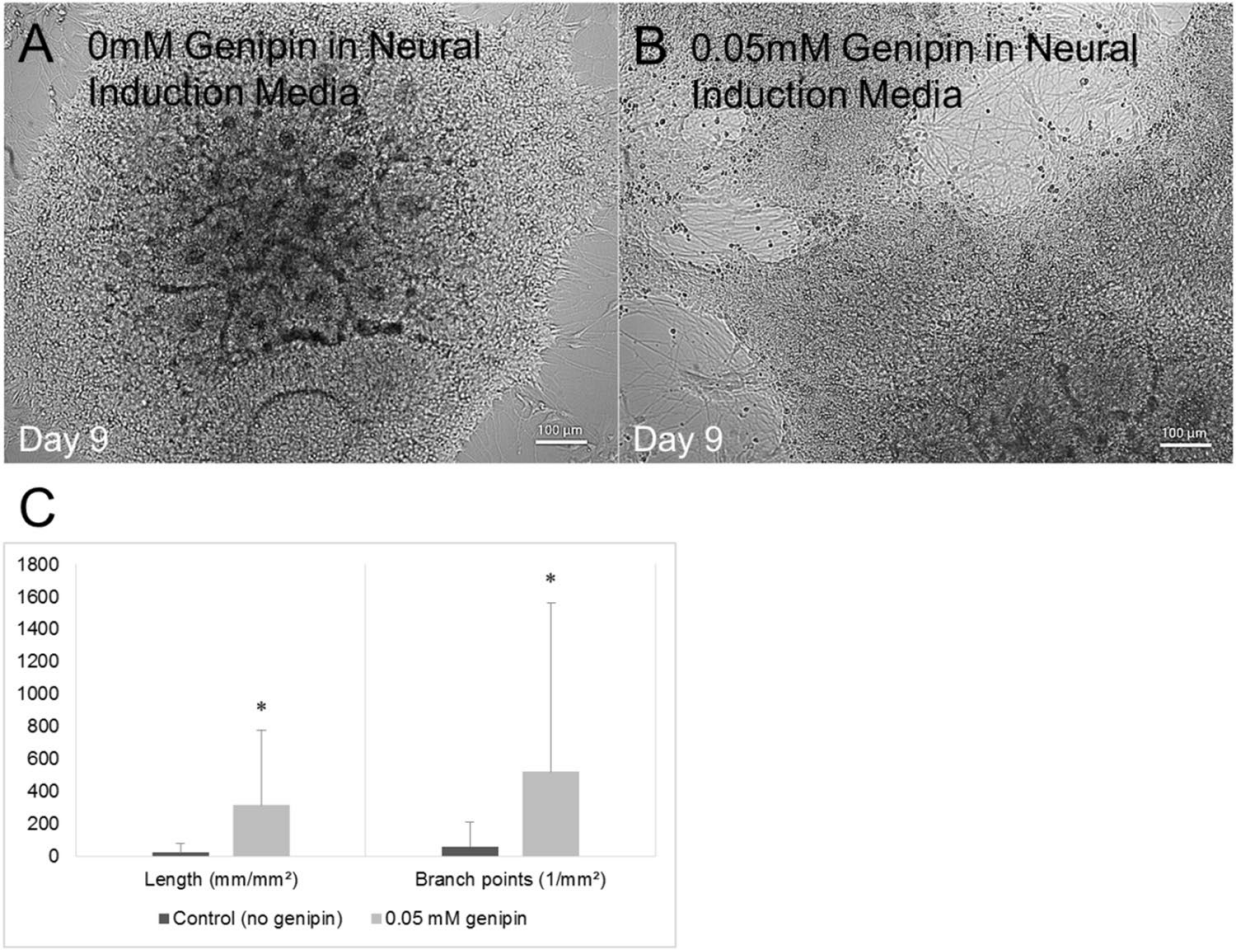
Genipin promotes neurite extension of human iPSC-derived neural progenitors cultured on 2D laminin surfaces. (**A**) Phase contrast image showing an hiPSC-derived neural aggregate with no genipin present. (**B**) Phase constrast image showing an hiPSC-derived neural aggregate treated with 0.05 mM genipin. (**C**) Quantification of the neurite length and branch points for each of the aforementioned groups. The group treated with 0.05 mM genipin showed increased neurite length and increased numbers of branch points, indicating more mature levels of differentiation. *Indicates p < 0.05 compared to control. Each group is represented by its mean average (technical n = 71 measurements) with error bars indicating the standard error of the mean. Each image represents a single neural aggregate with an initial seeding density of 4000 cells/aggregate (biological n = 3).

Based on the results of the cell viability analysis, the concentration of 0.05 mM was selected for further analysis of its effect on neural differentiation of hiPSC-derived neural aggregates. After 9 days, surviving neurites were measured and compared to a control group with no genipin added (Fig. 3). Both neurite length and branching were substantially greater in the genipin group compared to controls. This neuritogenic effect of genipin was previously observed in Neuro2A cells and PC12 cells as reported by Yamazaki *et al*. who hypothesized that genipin activates a nitric oxide(NO)-cyclic GMP(cGMP)-dependent protein kinase signaling pathway followed by activation of extracellular signal-regulated kinase (ERK)^30, 31^.

### Genipin cross-linking influences the mechanical properties and morphological properties of 3D fibrin scaffolds

**Figure 4:**
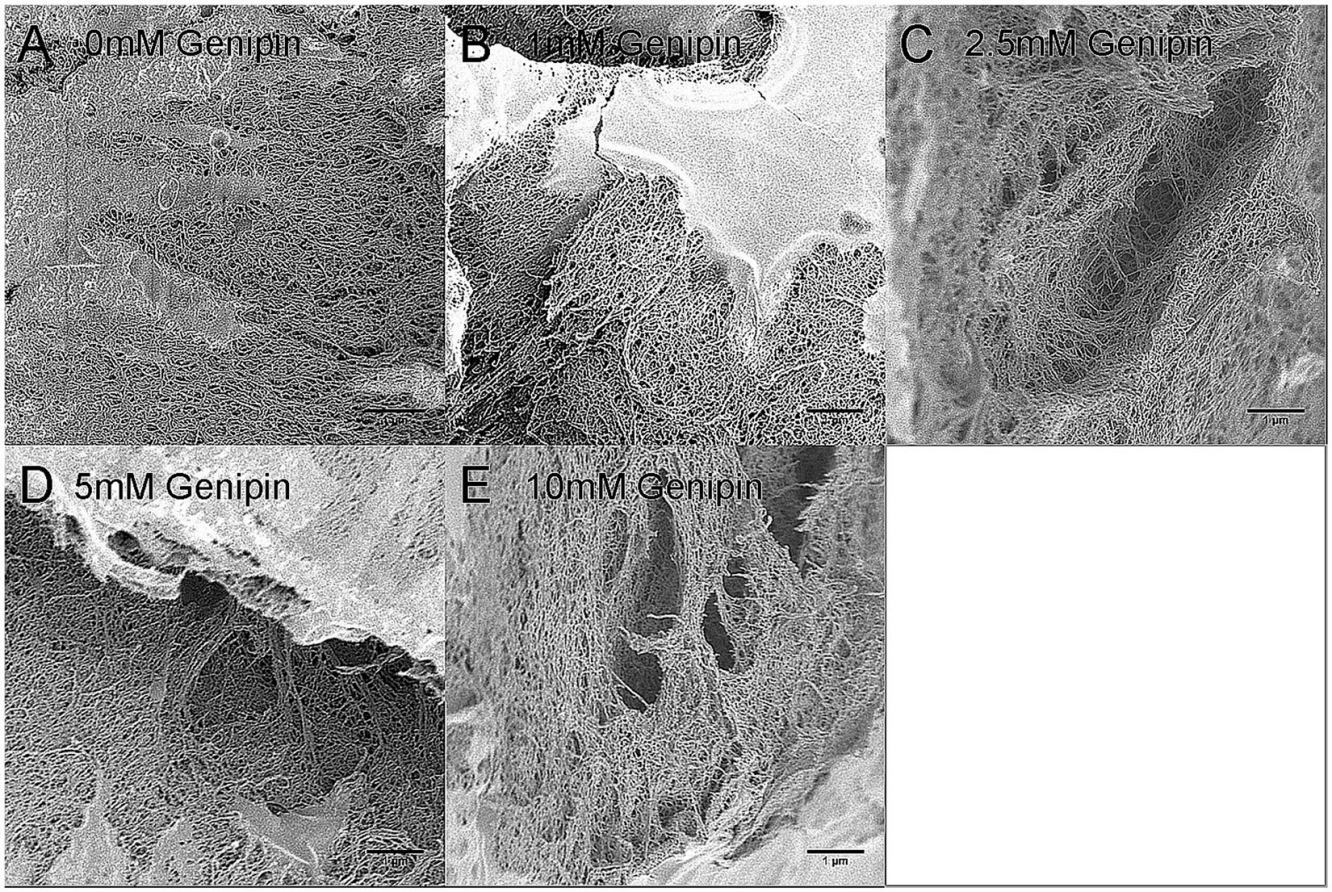
Scanning electron microscopy images of fibrin scaffolds crosslinked with the following concentrations of genipin: (**A**) 0 mM (**B**) 1 mM (**C**) 2.5 mM (**D**) 5 mM and (**E**) 10 mM. Images are representative of 3 samples taken (biological n = 3, technical n = 3).

Fibrin scaffolds were crosslinked using the following genipin concentrations: 1 mM, 2.5 mM, 5 mM and 10 mM. This range was chosen based on literature, which concluded that crosslinked fibrin using genipin in which 5 mM resulted in the slowest degradation rate but also exhibited toxicity to some cell types. 1 mM of genipin was seen by scanning electron microscopy to have similar fiber density and pore size to fibrin matrices without crosslinking (Fig. 4)^25, 28^.

**Figure 5:**
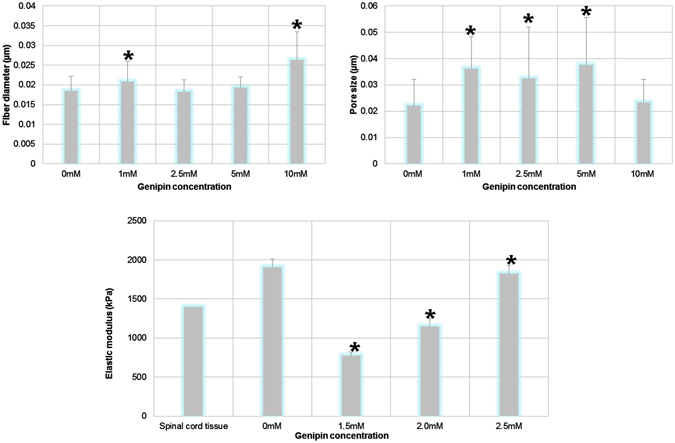
Genipin crosslinking influences the morphological and mechanical properties of fibrin scaffolds. (**A**) The effect of genipin concentration on fiber diameter (biological n = 3, technical n = 10). (**B**) The effect of genipin concentration on pore size of fibrin scaffolds (biological n = 3, technical n = 30). (**C**) The effect of genipin on elastic modulus. The elastic modulus for spinal cord tissue is taken from^37^. Each group is represented by its mean average with error bars indicating the S.E.M. *Indicates p < 0.05 compared to control (0 mM).

The scaffolds were characterized for their morphology and mechanical properties. The density of the fibrous matrix increases with higher concentrations of genipin, while pore sizes decreased (Fig. 5). The pore size increased with the addition of genipin and stayed pretty consistent until an increase in fiber size was noted at 10 mM, which corresponded to a decrease in pore size. Another group had observed similar effects and hypothesized that small precipitates of genipin prevent the fibrinogen chains from properly interacting with thrombin during polymerization^28^. This effect could also explain why the elastic modulus initially decreases with the addition of genipin before increasing. Additionally, the scaffolds appeared blue. The color intensified with increased crosslinking caused by higher concentrations of genipin, which was consistent with the aforementioned study^28^. Furthermore, mechanical testing of the scaffolds showed that fibrin crosslinked with 2.5 mM genipin had compressive moduli similar to that of spinal cord tissue^37^.

### Genipin crosslinked fibrin scaffolds do not induce death of hiPSC-derived neural progenitors

**Figure 6:**
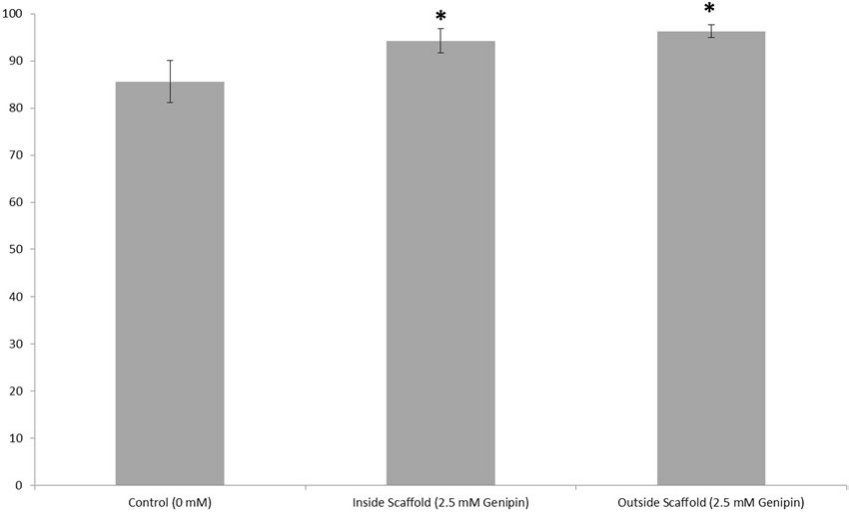
3D fibrin scaffolds crosslinked with genipin do not induce death of hiPSC-derived neural aggregates. Quantification of viability for hiPSC-derived neural progenitors seeded inside of 2.5 mM genipin-crosslinked scaffolds and beneath them in dual culture systems (biological n = 3, technical n = 5, data reported is mean ± standard error of the mean) *indicates p < 0.5 compared to the control.

To assess the effects of soluble genipin released from 3-D fibrin scaffolds, the lowest concentration that did not degrade within 14 days, 2.5 mM, was tested in a dual culture system where 3D fibrin-genipin scaffolds containing a single neural aggregate were suspended above 2D laminin cell cultures and soluble genipin was released into the media as the fibrin-genipin scaffold degraded. The effect of the soluble genipin was then noted by quantifying the viability of the cells inside the scaffolds and in 2D laminin cell cultures after 8 days, as shown in Fig. 6. Non-toxic levels of soluble genipin were leaked into the media for the 2.5 mM genipin-fibrin scaffolds. Furthermore, the observed increase in viability, in comparison to controls, suggests that the quantity of genipin released is less than soluble genipin at 0.05 mM, which promotes these neurotrophic effects (see Fig. 2).

### hiPSC-derived neural progenitors extend neurites inside of mechanically stable 3D fibrin scaffolds

**Figure 7:**
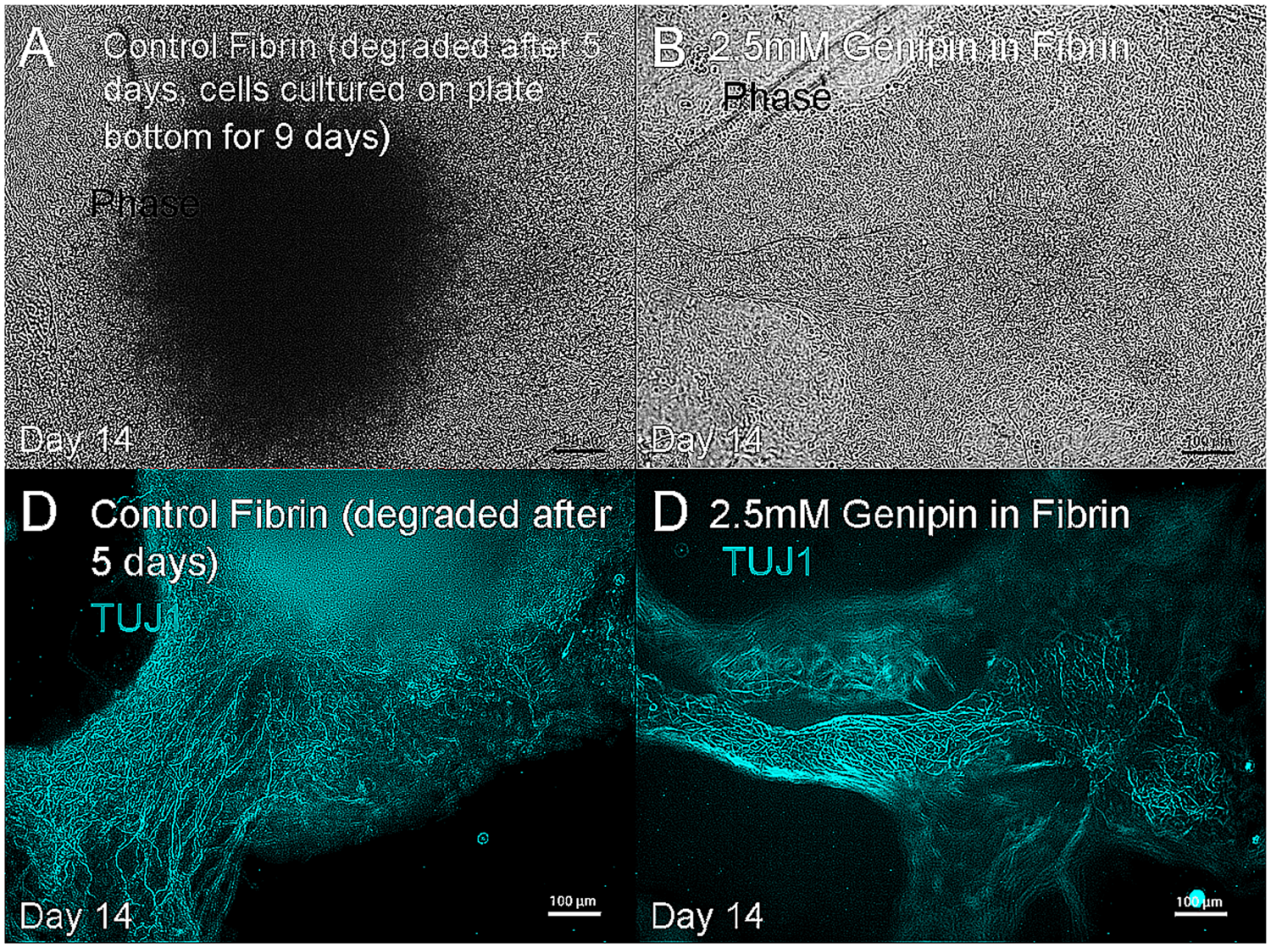
hiPSC-derived neural progenitors differentiate into neurons when seeded inside of mechanically stable genipin cross-linked fibrin scaffolds. (**A**) Neural aggregates seeded in fibrin degraded the fibrin within 5 days and attached to the plate bottom, whereas (**B**) neural aggregates seeded in 2.5 mM fibrin-genipin scaffolds differentiated within the 3-D scaffold which remained intact for 2 weeks. Each image represents a single neural aggregate with an initial seeding density of 4000 cells/aggregate. Image D is representative of 10 out of 24 biological replicates, which correlates with the percentage of neural aggregates showing signs of differentiation at the time of seeding.

We hypothesized that the lack of neurite outgrowth observed in the 3D fibrin-genipin scaffolds may be due to a deficiency in the intrinsic ability of neural aggregates to extend neurites when seeded in a 3D scaffold. Accordingly, we modified our protocol by extending the time the neural aggregates spent in Aggrewells from 5 to 7 days, changing the media 75% every other day instead of daily. We anticipated this process would allow for the necessary intercellular signaling to promote differentiating neural aggregates by the time of seeding. By day 7, it was noted that approximately 50% of the neural aggregates were exhibiting signs of differentiation. When seeded in fibrin scaffolds crosslinked with genipin using a lower range of concentrations from 1.5 mM to 2.5 mM, the neural aggregates continued to differentiate and extend neurites into the scaffolds for 14 days, as evidenced by staining for TUJ1 (Fig. 7). Neural aggregates were chosen from the Aggrewells at random for seeding with ~50% of those seeded exhibiting further differentiation and neurite extension. None of the genipin-crosslinked scaffolds exhibited signs of degradation by day 14. In contrast, fibrin scaffolds without crosslinking by genipin degraded completely by day 5, leaving the NAs to differentiate in 2D on the surface of the plate wells, where they over-proliferated and showed signs of necrotic centers by day 14. Fibrin crosslinked with 2.5 mM genipin appeared to encourage the most infiltration into the scaffolds, as seen by TUJ1 staining. It is also possible based on our earlier results that more cells survived at the lower concentration, enabling more neurite extension.

**Figure 8:**
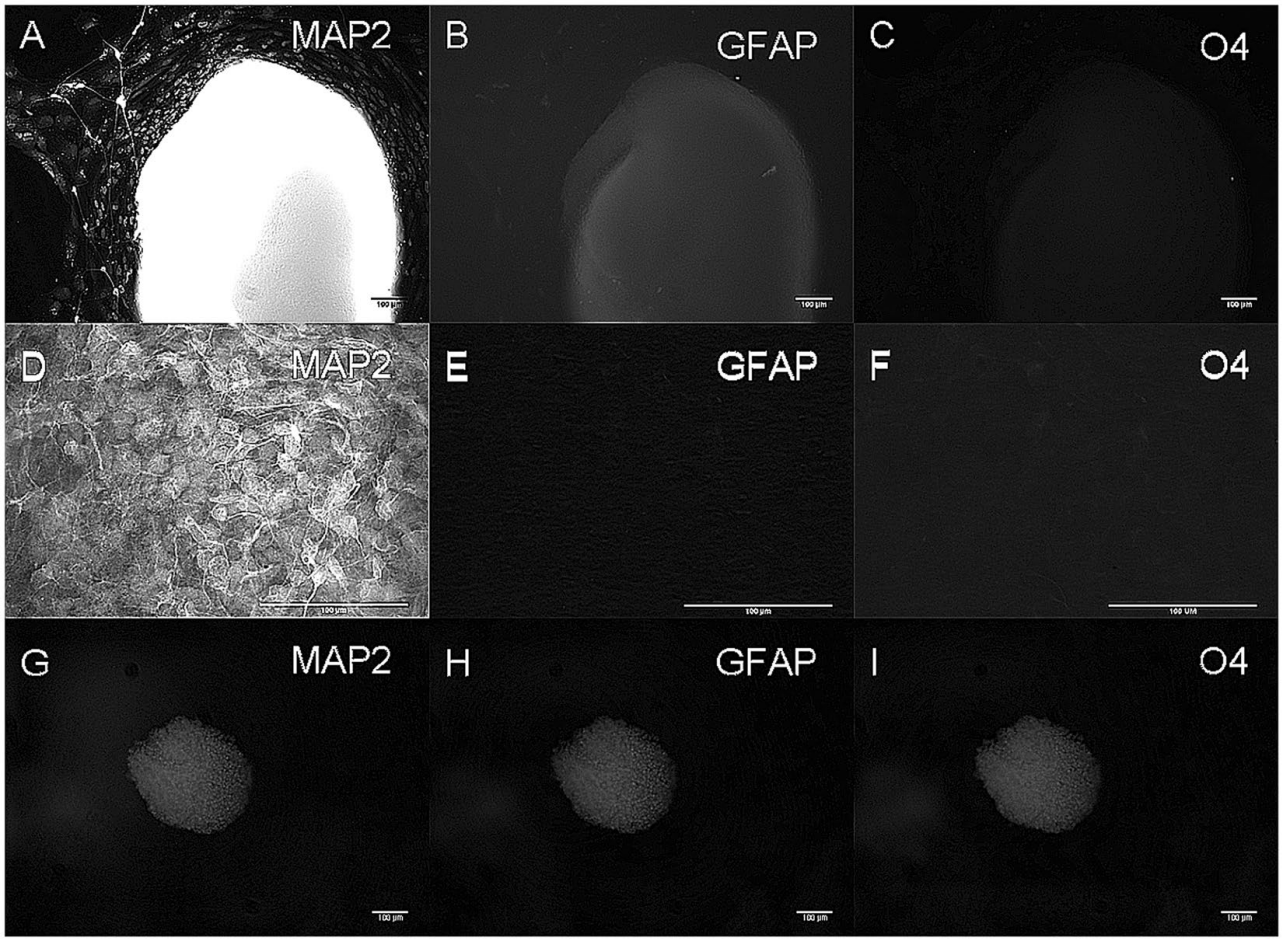
hiPSC-derived aggregates do not express the astrocyte marker GFAP and oligodendrocyte marker O4. (**A**–**C**) Neural aggregates seeded in fibrin are positive for mature neuronal marker MAP2 but not GFAP or O4. (**D**–**E**) High magnification image of (**A–C**). (**G–I**) Neural aggregates in genipin-crosslinked fibrin are negative for MAP2, O4 and GFAP. Each image represents a single neural aggregate with an initial seeding density of 4000 cells/aggregate (biological n = 3, technical n = 3).

Neural aggregates were negative for oligodendrocyte and astrocyte markers O4 and GFAP as well as the mature neuronal marker MAP2, indicating that after two weeks the neural aggregates were composed of immature neurons. Control neural aggregates grown in fibrin were also negative for O4 and GFAP but were positive for MAP2 suggesting that genipin crosslinking may affect their rate of maturation (Fig. 8).

The development of a stable fibrin formulation that supports neurite outgrowth is an exciting development that extends the potential of this commonly used biomaterial. In particular, this fibrin formulation has potential for being translated into a bioink suitable for 3D printing as fibrin has been a challenging biomaterial to print^38^.

## Conclusions

Our findings were consistent with previous reports of genipin in terms of crosslinking, morphology, mechanical properties, cell toxicity and neurite-promoting effects^24, 26, 27, 30, 31, 33^. Genipin at low concentrations was non-toxic and enhanced neurite outgrowth in neural aggregates derived from hiPSCs. At higher concentrations, genipin slowed degradation of fibrin matrices by cells while inhibiting neurite outgrowth and causing cytotoxicity. The highest genipin concentrations induced cell death. Genipin concentrations between 1 mM and 2.5 mM showed that there is an optimal concentration where the neurotrophic and fibrin cross-linking effects of genipin co-exist. This work also further extends the utility of genipin for the use of engineering tissues from human pluripotent stem cells. A recent study demonstrated that genipin could be used to crosslink decellularized extracellular matrix secreted by mouse neural progenitor cells derived from embryonic stem cells to form stable scaffolds^39^. However, this paper is the first to report its effects on human cells. Additionally, the use of genipin is also compatible with 3D printers and this work may serve as a starting point for formulating stable bioinks for printing hiPSC-derived neural cells into tissues.

## Methods

### Culture and differentiation of hiPSCs

All work involving human iPSCs was done with approval from the University of Victoria’s Human Ethics committee. WiCell human foreskin1 iPSCs were obtained from the Lab of Dr. James Thomson at the University of Wisconsin. iPSC identity was authenticated by STR testing by UW Molecular Diagnostics Laboratory using a Promega PowerPlex1.2 System. iPSCs tested negative for mycoplasma contamination by a Bionique M250 test. Stem cell culture media and regents were purchased from STEMCELL Technologies with the exception of laminin and poly-L-ornithine from Sigma. Human induced pluripotent stem cells were cultured in defined conditions and differentiated into neural aggregates as previously described^21, 40^. For the final experiment, the neural aggregates were allowed to form for 7 days in the Aggrewell. These neural aggregates were then either plated on 2-D poly-L-ornithine/laminin-coated surfaces or seeded into 3-D genipin-fibrin crosslinked scaffolds in the presence of 1 μM soluble retinoic acid, 1 µM soluble purmorphamine^41^ and genipin when indicated. In the experiments with soluble genipin, neural aggregates were seeded on poly-L-ornithine/laminin coated plates with a range of genipin concentrations in Neural Induction Media (STEMCELL Technologies).

### Analysis of cell behavior

Neurite length and branch points were quantified using an IncuCyte Neurotracker software as detailed previously^42, 43^. Neurite length and branch points were quantified using IncuCyte Neurotracker software. The cells were imaged in by an IncuCyte ZOOM^R^, a live-content imaging platform from Essen BioScience, which comes with a software module called Neurotrack^R^ that uses an algorithm to mask and analyse neurite length and branching over time. It has been validated for a variety of neuronal types with a sensitivity equivalent to that of an immunofluorescence-based, high content imaging assay. Immunohistochemistry for the neuronal maker Tuj1 (Millipore MAB1637), O4 (Millipore MAB345), Map2 (Abcam ab3592), and GFAP (Abcam ab7260) were performed as previously described^41^. For cell viability staining, 4 μm of ethidium homodimer solution (Invitrogen Molecular Probes L3224) was added to cell cultures, and allowed to incubate for 30 minutes before imaging. Cells were imaged using a Leica DMI 30000B microscope with a QImaging RETIGA 2000R camera at 100× magnification. Cell death was quantified by flow cytometry using Guava Viacount assay (Millipore).

### Formation and characterization of genipin-fibrin crosslinked scaffolds

All chemicals mentioned in this section were purchased from Sigma unless otherwise noted. Fibrin scaffolds (final concentration: 10 mg/mL) were prepared as previously described^21, 41^. Genipin diluted in phosphate buffered saline was then added to the polymerized fibrin scaffolds and incubated at 37 °C and 5% CO_2_ in the dark for 18 hours to allow for crosslinking. Scaffolds were then rinsed thoroughly with phosphate buffered saline. Crosslinked fibrin samples were compressed while force and displacement were measured continuously by electronic sensors (Interlink Electronics) and a linear motion potentiometer (Bourns) to determine the elastic modulus. Average radius and height of the samples were obtained using digital calipers (Mitutoyo Corporation) (n = 3). Stress and strain were then calculated and linear regression was used to determine the elastic moduli of the samples. Standard error of the slope was determined using Minitab Statistics software with a confidence interval of 95%. For analysis using scanning electron microscopy, genipin-fibrin crosslinked scaffolds (n = 3) were fixed with 2.5% glutaraldehyde solution followed by dehydration through an ethanol series, and critical point drying. Samples were then mounted onto stubs and sputter coated with 1–2 nm gold and imaged^44^. Fiber diameter and pore size was assessed using ImageJ as done previously^45^.

### Statistical Analysis

Significance was determined first by ANOVA using Excel, then in Minitab software by computing two-tailed student t-tests with α = 0.05. Elastic moduli curves were fitted to regression models in Minitab software to compute slopes and standard errors. For cultures in 2-D, a sample size of 3 neural aggregates for each group, each with a starting population of approximately 4000 cells, and each exposed to the various conditions for 7–9 days, was considered adequate since neural aggregates of this size are known to reliably expand and form neural rosettes in 7–9 days when plated on a 2-D laminin substrate (see Stemcell Technologies Aggrewell Manual). The expansion and differentiation capability of neural aggregates in 3-D genipin-fibrin gels were not known so the sample size for this experiment was n = 24 to better correlate the observable effects with each experimental group. In the neurite metrics analysis, one neural aggregate in the control group was deemed to be an outlier in comparison to the other control samples and excluded. In all cases, variance estimated by S.E.M was found to be within 2% of the mean for each group.
